# A shortened uncemented stem offers comparable positioning and increased metaphyseal fill compared to a standard uncemented stem

**DOI:** 10.1186/s40634-019-0197-1

**Published:** 2019-06-25

**Authors:** Alexandre Jacquel, Augustin Le Viguelloux, Jeremy Valluy, Mo Saffarini, Nicolas Bonin

**Affiliations:** 1 Ramsay Générale de Santé, Clinique de la Sauvegarde, Lyon-Ortho-Clinic, 8 Avenue Ben Gourion, 69009 Lyon, France; 2 Centre Hospitalier William Morey, 4 Rue Capitaine Drillien, 71100 Chalon Sur Saône, France; 3 ReSurg SA, Rue Saint Jean 22, 1260 Nyon, Switzerland

**Keywords:** Total hip arthroplasty, THA, Shortened stems, Axial alignment, Canal-fill ratio, Metaphyseal fill

## Abstract

**Background:**

Shortened stems are increasingly used in uncemented total hip arthroplasty (THA) as they represent a compromise between the metaphyseal anchorage of short stems and the facilitated axial alignment of standard stems. The purpose of this study was to compare the metaphyseal canal-fill ratio (CFR) and axial alignment of a shortened double-tapered stem with those of a standard stem. The hypothesis was that the shortened stem would achieve greater metaphyseal fill and comparable axial alignment.

**Methods:**

The authors reviewed routine follow-up anteroposterior radiographs taken 2 months after THA to evaluate metaphyseal fill and axial alignment of a shortened stem (*n* = 96) and a standard stem (*n* = 101). The CFR was calculated at the level of the tip and superior margin of the lesser trochanter. Stem alignment was defined as the angle between the stem axis and the proximal anatomic femoral axis. Stems were classified as being in varus or valgus alignment if they deviated by more than 3° from the anatomic axis of the femur.

**Results:**

Hips implanted with shortened and standard stems had comparable demographics and axial alignment (1.1° ± 1.7° vs 0.8° ± 1.2°; *p* = 0.331). However, varus alignment was observed in 5% of shortened stems compared to only 1% of standard stems, though this difference was not significant (*p* = 0.111). The femoral CFR was greater using shortened stems than using standard stems, both at the level of the tip of the lesser trochanter (0.91 ± 0.05 vs 0.85 ± 0.08; *p* < 0.001) and at its superior margin (0.76 ± 0.06 vs 0.72 ± 0.07; *p* < 0.001).

**Conclusions:**

Compared to the standard stem, the shortened stem had increased metaphyseal filling and equivalent alignment. These findings suggest that shortened stems could provide adequate metaphyseal fixation and correct alignment. Further studies remain necessary to evaluate how shortened stems perform in terms of osseointegration, clinical outcomes and survival.

## Background

Total hip arthroplasty (THA) using uncemented femoral stems demonstrated excellent long-term fixation (Khanuja et al., 2011) and clinical results (Tannast et al., 2009), though it remains associated with mid-thigh pain (Jo et al., 2016; Mihalko & Whiteside, 2015; Petis et al., 2015) likely related to inadequate load transfer (Fottner et al., 2018), and stress-shielding due to predominantly diaphyseal fixation (Khanuja et al., 2011). The latter is often combined with inadequate metaphyseal loading, indicated by a low canal-fill ratio (CFR) at the level of the lesser trochanter, which is associated with poor osseointegration (Ishii et al., 2016) and thigh pain (Nam et al., 2015). In response to these problems, and with less invasive techniques, shorter uncemented femoral stems were introduced (Falez et al., 2015; Molli et al., 2012; Patel & Stulberg, 2014) to optimize load transfer and reduce stress-shielding, as well as preserve bone stock (Molli et al., 2012; Small et al., 2017; Yan et al., 2017).

Various designs of short femoral stems were introduced to increase metaphyseal loading (Falez et al., 2015; Khanuja et al., 2014). Partial neck-preserving or trochanter-sparing designs rendered promising early results but were later reported to exhibit poor alignment (Khanuja et al., 2014; Lombardi Jr. et al., 2009; Shishido et al., 2018) and primary instability (Giardina et al., 2018), which increases risks of stem loosening and revision (Krismer et al., 1999). Malalignment of short stems, particularly excessive varus, is believed to induce thigh pain (Amendola et al., 2017; Cinotti et al., 2013; Gielis et al., 2019), while excessive valgus is associated with initial stem subsidence (Kutzner et al., 2017; Mahmoud et al., 2017). Shortened diaphyseal stems were developed as a compromise between the metaphyseal anchorage of short stems and the facilitated axial alignment of standard stems. This design rendered good clinical outcomes in smaller patients (Choy et al., 2013; Feyen & Shimmin, 2014) and is being increasingly used for the general population. However, there are no published studies investigating its metaphyseal fill and axial alignment.

The purpose of this study was therefore to compare the CFR and axial alignment of standard and shortened uncemented femoral stems. The hypothesis was that, compared to the standard stem, the shortened stem would achieve greater metaphyseal fill and comparable axial alignment.

## Methods

Between May 2015 and April 2016, the senior author performed 217 primary THAs using two different uncemented femoral stems in roughly equal proportions. During this period, the choice of stem depended on available stock at hospital inventory, rather than surgeon preferences or patient characteristics:

1. The Hype SCC standard stem (SERF, Décines-Charpieu, France) was implanted in 110 hips (50.7%); the surgeon was familiar with this stem and its instrumentation as he had been implanting it for 3 years. The Hype stem length ranges from 135.7 mm (offset 38 mm) to 193 mm (offset 48 mm);
2. The Symbol Std HA Collared shortened stem (Dedienne Santé, Nimes, France) was implanted in 107 hips (49.3%); the surgeon was in his learning curve with this stem and its instrumentation as the system had just been introduced. The symbol stem length ranges from 105.3 mm (offset 38 mm) to 151.3 mm (offset 48 mm).

**Fig. 1:**
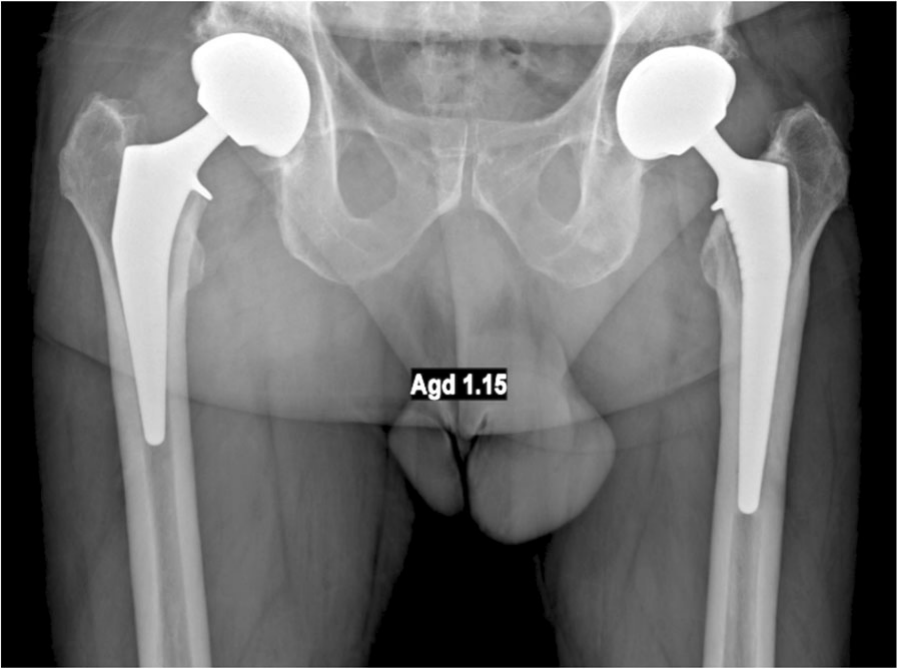
Postoperative anteroposterior radiograph of both implants, implanted in the same patient. The Symbol (shortened stem) is on the left and the Hype (standard stem) on the right

The design of the Symbol stem is comparable to that of the Hype stem, except for shortening of the stem length by approximately 30% and a slight increase of anteroposterior dimension by 6%–9%. Both stems have a dual-tapered geometry and are hydroxyapatite-coated on their entire intra-medullary surface (Fig. 1).

The authors reviewed routine follow-up anteroposterior radiographs taken 2 months after surgery and investigated stem metaphyseal filling and axial alignment. Due to insufficient quality or inadequate magnification, 9 hips implanted with the standard stem and 11 hips implanted with the shortened stem were excluded. This left radiographs of 101 hips implanted with the standard stem and of 96 hips implanted with the shortened stem.

**Fig. 2:**
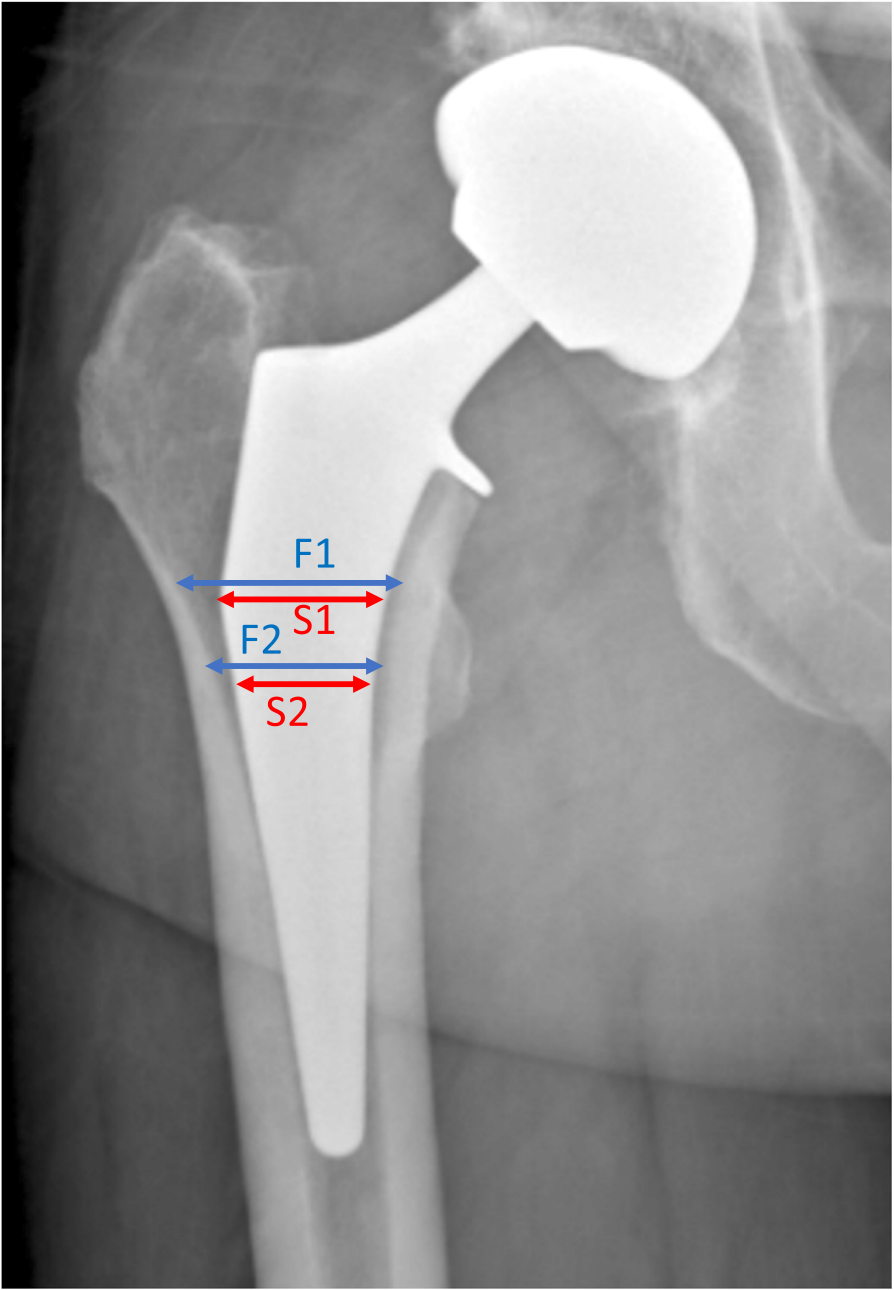
Postoperative radiograph detailing the measurements of canal fill ratio. The blue lines represent the measurement of the width of the inner femoral cortex (F1), and the red lines those of the stem width (S1). The CFR is the ratio between the width of the stem and that of the inner femoral cortex (S1/F1). It is measured at both the upper edge of the lesser trochanter (F1/S1), and at its tip (F2/S2)

**Fig. 3:**
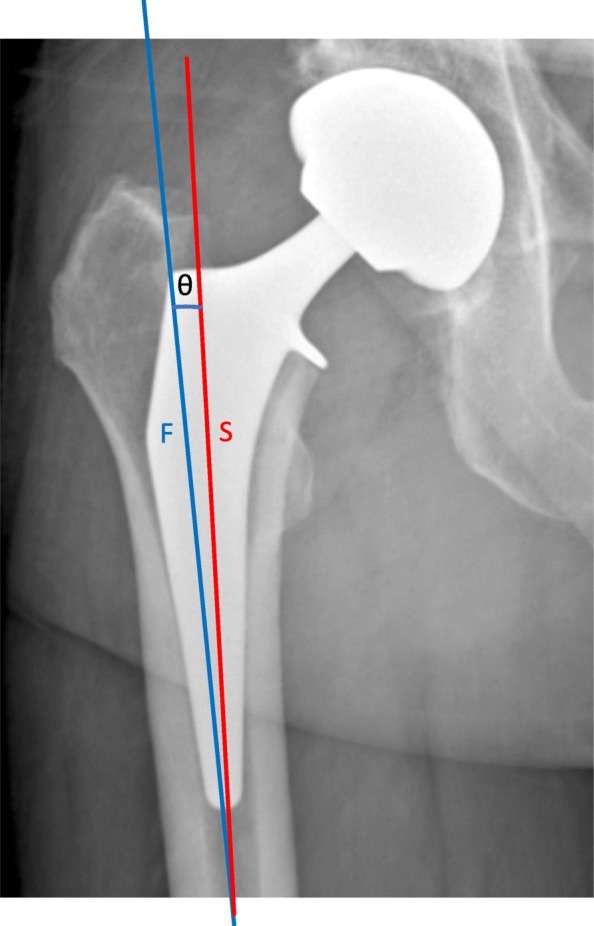
Postoperative radiograph detailing the measurements of stem axial alignment, that is the angle (θ) between the stem axis (S, red; indicated on templates provided by the manufacturer) and the proximal femoral axis (F, blue)

The radiographs were used to calculate the canal-fill ratio, defined as the width of the stem divided by the width of the inner femoral cortex at 2 points: at the tip of the lesser trochanter (LT), and at the superior margin of the LT (Fig. 2), as well as the Canal Flare Index (CFI) (Noble et al., 1988), defined as the ratio of the diameter of the femoral canal at the isthmus to the diameter of the medullary canal 20 mm above the lesser trochanter. The radiographs were also used to evaluate stem alignment, that is the angle between the stem axis (indicated on templates provided by the manufacturer) and the proximal femoral axis (a line passing through the center of the intramedullary canal at the isthmus and 20 mm proximal to the LT) (Noble et al., 1988) (Fig. 3). Stems were classified as being in varus or valgus alignment if they deviated by more than 3° from the proximal axis of the femur (Gromov et al., 2017). The inter-observer repeatability of the radiographic measurements was determined by having a second surgeon independently re-measure a random selection of 30 radiographs using a standardized protocol. The intra-class correlation coefficient was good (0.81) for stem alignment and moderate (0.50 and 0.60) for the CFR (Table 1) (Koo & Li, 2016). The moderate inter-observer agreement for the CFR is likely due to the difficulty in determining the precise borders of the femoral cortex on the radiographs.

**Table 1:** Inter-observer reliability (intra-class coefficient)

	Inter-observer		
	*ICC*	95% C.I.	
Canal-fill ratio (CFR)			
Superior margin of the Lesser Trochanter	0.60	(0.20	– 0.81)
Tip of the Lesser Trochanter	0.50	(0.11	– 0.74)
Stem alignment	0.81	(0.64	– 0.91)

All radiographs used were taken for routine postoperative follow-up and the patients were not subjected to additional radiographs for the purpose of this study. All patients gave written consent for the use of their data and images for research and publication purposes.

### Statistical analysis

A priori power analysis determined that 23 patients in each cohort would provide a sufficient power (alpha = 0.05; beta = 0.90) to detect a difference in mean axial alignment of 2° between the standard and shortened stems, assuming standard deviations of 2°. Descriptive statistics were used to summarize the data. The cohorts were compared for baseline preoperative data (age, Body-Mass-Index (BMI), CFI) and intra-operative data pertaining to the implants (stem size, neck type, presence of a collar), as well as radiographic measurements (CFR and axial alignment). Shapiro-Wilk tests were used to assess the normality of distributions. For non-Gaussian quantitative data, differences between groups were evaluated using the Wilcoxon rank sum test (Mann Whitney U test). For categorical data, differences between groups were evaluated using Fisher’s exact test. Statistical analyses were performed using R version 3.3.2 (R Foundation for Statistical Computing, Vienna, Austria). *P*-values < 0.05 were considered statistically significant.

## Results

There were no differences between the standard and shortened stems in terms of patient age (63.4 ± 14.1 vs 62.8 ± 10.6; *p* = 0.290), sex (47% women vs 45%; *p* = 0.916) and BMI (27.7 ± 6.3 vs 26.0 ± 4.0; *p* = 0.107) (Table 2). All shortened stems were collared, compared to only 78% of standard stems (*p* < 0.001), because the high-offset model of the standard (Hype) stem was only available in the collarless version (Table 3). However, there were no differences between patients who had collared and collarless standard stems in terms of CFR at the tip of the lesser trochanter (0.83 ± 0.09 vs 0.85 ± 0.07, *p* = 0.357) and at its superior margin (0.71 ± 0.07 vs 0.72 ± 0.07, *p* = 0.790), as well as stem mal-alignment (0% vs 1%, *p* = 0.202).

**Table 2:** Patient demographics

	Standard stem (*n* = 101)		Shortened stem (*n* = 96)		
	*mean ± SD*	*(range)*	*mean ± SD*	*(range)*	*p*-value
Age	63.4 ± 14.1	(19.0–92.0)	62.8 ± 10.6	(36.0–85.0)	0.290
BMI	27.7 ± 6.3	(18.4–56.8)	26.0 ± 4.0	(18.6–40.8)	0.107
Canal Flare Index (CFI)	3.6 ± 0.6	(2.1–4.8)	3.7 ± 0.7	(2.3–5.1)	0.310
Women	47 (47%)		43(45%)		0.916

**Table 3:** Stem characteristics

	Standard stem (*n* = 101)	Shortened stem (*n* = 96)	*p*-value
Stem size			
2	6(6%)	4(5%)	
3	30(29%)	7(8%)	
4	27(28%)	13(14%)	
5	20(20%)	19(20%)	
6	14(12%)	22(23%)	
7	2(2%)	18(19%)	
8	0(0%)	7(8%)	
9	0(0%)	4(3%)	
10	0(0%)	2(3%)	
11	2(2%)	0(0%)	
Neck Type			0.063
Standard	77(77%)	84(89%)	
Lateralized	24(23%)	12(11%)	
Collar	78(78%)	96(100%)	< 0.001

**Fig. 4:**
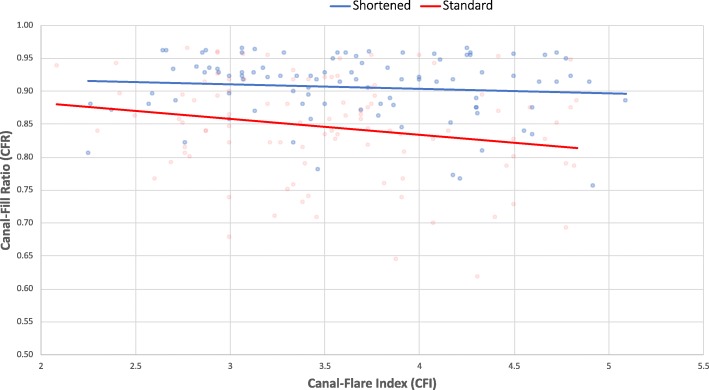
Scatterplot of the canal-fill ratio (CFR) of the shortened and standard stems at the level of the tip of the lesser trochanter, in relation to the patients’ canal-flare index (CFI)

The CFR was significantly greater at the tip of the LT for the shortened stems (0.91 ± 0.05) compared to the standard stems (0.85 ± 0.08) (*p* < 0.001) (Table 4). This was true regardless of femur morphology, with an increasing CFR difference for patients with higher CFI (Fig. 4). The CFR was likewise greater at the superior margin of the LT for the shortened stems compared to the standard stems (0.76 ± 0.06 vs 0.72 ± 0.07; *p* < 0.001).

**Table 4:** Stem filling and positioning

	Standard stem (*n* = 101)		Shortened stem (*n* = 96)		
	*mean ± SD*	*(range)*	*meana ± SD*	*(range)*	*p*-value
Canal-Fill Ratio (CFR)					
Superior margin of the Lesser Trochanter	0.72 ± 0.07	(0.55–0.90)	0.76 ± 0.06	(0.59–0.91)	< 0.001
Tip of the Lesser Trochanter	0.85 ± 0.08	(0.62–0.96)	0.91 ± 0.05	(0.77–0.96)	< 0.001
Stem alignment (°)	0.8 ± 1.2	(−2–3)	1.1 ± 1.7	(−3–7)	0.331
Valgus (<−3°)	0(0%)		0(0%)		0.232
Varus (> 3°)	1(1%)		5(5%)		0.111

There was no significant difference in axial alignment of the shortened stems compared to the standard stems (1.1° ± 1.7° vs 0.8° ± 1.2°; *p* = 0.331) (Table 4). It is worth noting, however, that varus alignment > 3° was observed in 5% of shortened stems compared to only 1% of standard stems, though this difference was not significant (*p* = 0.111).

The authors investigated the potential learning curve bias in the shortened stem cohort, and found no significant differences between the first and last quintiles of patients operated in terms of CFR at the tip (0.91 ± 0.04 vs 0.91 ± 0.03, *p* = 0.960) and superior margin (0.74 ± 0.06 vs 0.76 ± 0.07, *p* = 0.117) of the lesser trochanter. It is interesting to note that none of the mal-aligned stems was implanted in the first quintile of patients operated.

## Discussion

The main finding of this study is that the novel shortened stem is more filling in the metaphysis than the standard stem, and equally well aligned. However, the higher number of outliers in varus for the shortened stem could warrant further scrutiny. Although we found no significant difference in alignment between the two stems, our sample size may have been insufficient to conclude.

While some authors warned about the consequences of varus alignment on load transfer (Fottner et al., 2018) and thigh pain (Gielis et al., 2019), and of those of valgus malalignment on stem early subsidence (Kutzner et al., 2017), it remains unclear how much stem malalignment can lead to adverse events or compromise clinical outcomes (de Beer et al., 2006; Lombardi Jr. et al., 2009; Shishido et al., 2018). While several authors found that varus-aligned short stems are associated with poorer outcomes, including thigh pain and primary instability (Drosos & Touzopoulos, 2019; Giardina et al., 2018), this does not seem to impact long-term outcomes and overall complication rates (Giardina et al., 2018; Wacha et al., 2018). Taken together, these findings nevertheless suggest that correct alignment of short stems is important to improve primary stability and thigh pain after THA. With their similar metaphyseal design to highly successful standard stems, shortened stems might be an effective compromise between shorter length and adequate alignment.

For uncemented stems, metaphyseal fill is very important to minimize stress shielding and achieve physiological load transfer (Huiskes & van Rietbergen, 1995; Weinans et al., 1994). In theory, reducing the length of the stem reduces stress shielding, as contact between the stem in the distal cortex is minimized (Kim et al., 2001), though this has been debated in dual-energy X-ray absorptiometry studies (Lerch et al., 2012). In 2016, Ishii et al. (Ishii et al., 2016) revealed that the relation between proximal and distal fill was an important factor determining radiological changes after THA, particularly for femurs with narrow and flared femoral canals. They found that insufficient canal filling at the level of the LT was associated with failed osseointegration. Moreover, (Nam et al., 2015) stems with high distal CFR and low proximal CFR were shown to be associated with thigh pain after THA. In the present study, we showed that unlike the standard stem, the shortened stem offered consistently high CFR at the level of the LT regardless of femoral morphology, which could make them particularly suited to patients with flared femoral canals. However, we did not investigate the diaphyseal fill in the present study and can draw no definite conclusions. Stem design also affects bone resorption patterns, though the literature is conflicted as to the effect of stem length on bone resorption (Arno et al., 2012; Bieger et al., 2012; Boyle & Kim, 2011; van Rietbergen & Huiskes, 2001). There is therefore a possibility that the intermediate length of shortened stems provides an adequate balance between metaphyseal and distal stability, although no studies have yet investigated bone resorption patterns using this design. Finally, short uncemented stems are associated with significantly reduced primary stability (Ong et al., 2009), and although our results are encouraging, they cannot provide a definite answer as to the primary stability of shortened stems. A recent study evaluating early outcomes of THA using a comparable shortened stem to the one evaluated in this study, but collarless, found encouragingly low rates of stem subsidence, which exhibited no progression after 6 months (Attenello et al., 2019). It is nevertheless important to note that CFR alone cannot predict stem subsidence, good osseointegration or load transmission, so that further investigation remains necessary to evaluate the success of shortened stems.

In terms of classification of short stems, the shortened stem corresponds to the “trochanter-harming” category of Falez et al. (Falez et al., 2015). A recent classification (Drosos & Touzopoulos, 2019) introduced a category of “short versions of a standard stem”, which corresponds better to the stem used in this study. To the author’s knowledge, clinical results are still lacking in the literature for this type of stems. In agreement with Falez et al. (Falez et al., 2015), we expect outcomes in line with standard or trochanter-sparing implants, with potential added benefits of reduced thigh pain and subsidence. Clinical and radiographic studies are therefore warranted to assess outcomes of the shortened stems, in particular to evaluate whether this design is associated with a reduction in negative outcomes of uncemented THA, including stress shielding, thigh pain, and aseptic loosening.

This study confirmed that the design strategy of the shortened stems was successful in ensuring superior metaphyseal fill and comparable alignment to standard stems. However, the study has a number of limitations that must be acknowledged. First, the implants used were not perfectly equivalent, as all the shortened stems were collared, compared to only 78% of the standard stems, because the high-offset model of the latter is only available in the collarless version. Second, the inclusion period coincides with the surgeon’s learning curve of the shortened stems. Third, the CFR could only be assessed in the frontal plane as only anteroposterior radiographs were obtained. In addition, the CFR was not calculated below the LT, so we could not draw any conclusions on distal filling. Further, this radiographic study did not include an evaluation of clinical outcomes of surgery, and the short-term outcomes of the Symbol stem will be presented elsewhere. Finally, it is important to keep in mind that the present results were measured on two stem models and may not be applicable to other models or short stem designs.

## Conclusion

Compared to the standard stem, the shortened stem had increased metaphyseal filling regardless of femoral morphology, and equivalent alignment. These findings suggest that shortened stems could provide adequate compromise between sufficient stability owing to greater metaphyseal fill while providing correct alignment. Further studies remain necessary to evaluate how shortened stems perform in terms of clinical outcomes, osseointegration and survival.

## Data Availability

The datasets used and/or analyzed during the current study are available from the corresponding author on reasonable request.

## Abbreviations

BMI: Body-mass index
CFI: Canal-flare index
CFR: Canal-fill ratio
LT: Lesser Trochanter
THA: Total hip arthroplasty
